# Nuclear Factor AP2X-4 Governs the Expression of Cell Cycle- and Life Stage-Regulated Genes and is Critical for *Toxoplasma* Growth

**DOI:** 10.1128/spectrum.00120-22

**Published:** 2022-06-23

**Authors:** Jingwen Zhang, Fuqiang Fan, Lihong Zhang, Bang Shen

**Affiliations:** a State Key Laboratory of Agricultural Microbiology, College of Veterinary Medicine, Huazhong Agricultural University, Wuhan, Hubei Province, People’s Republic of China; b Key Laboratory of Preventive Medicine in Hubei Province, Wuhan, Hubei Province, People’s Republic of China; c Hubei Hongshan Laboratory, Wuhan, Hubei Province, People’s Republic of China; Weill Cornell Medicine

**Keywords:** AP2 factor, AP2X-4, virulence, rhoptry neck protein, invasion, bradyzoite differentiation cell cycle

## Abstract

Toxoplasma gondii is a ubiquitous pathogen infecting one third of the world’s population and diverse animals. It has a complex life cycle alternating among different developmental stages, which contributes to its transmission and pathogenesis. The parasite has a sophisticated gene regulation network that enables timely expression of genes at designated stages. However, little is known about the underlying regulatory mechanisms. Here, we identified an AP2 family transcription factor named TgAP2X-4, which was crucial for parasite growth during the acute infection stage. *TgAP2X-4* deletion leads to reduced expression of many genes that are normally upregulated during the M phase of the cell cycle. These include genes that encode rhoptry neck proteins that are key for parasite invasion. As a result, the Δ*ap2X-4* mutant displayed significantly decreased efficiency of host cell invasion. Transcriptomic analyses suggested that TgAP2X-4 also regulates a large group of genes that are typically induced during chronic infection, such as *BAG1* and *LDH2*. Given the diverse impacts on gene expression, TgAP2X-4 inactivation results in severely impaired parasite growth, as well as drastic attenuation of parasite virulence and complete inability to form chronic infection. Therefore, TgAP2X-4 represents a candidate for antitoxoplasmic drug and vaccine designs.

**IMPORTANCE** Toxoplasma gondii has a complicated gene regulation network that allows “just in time” expression of genes to cope with the physiological needs at each stage during the complex life cycle. However, how such regulation is achieved is largely unknown. Here, we identified a transcription factor named TgAP2X-4 that is critical for the growth and life cycle progression of the parasite. Detailed analyses found that TgAP2X-4 regulated the expression of many cell cycle-regulated genes, including a subset of rhoptry genes that were essential for the parasites to enter host cells. It also regulated the expression of many genes involved in the development of chronic infection. Because of the diverse impacts on gene expression, TgAP2X-4 inactivation caused reduced parasite growth *in vitro* and attenuated virulence *in vivo*. Therefore, it is a potential target for drug or vaccine designs against *Toxoplasma* infections.

## INTRODUCTION

Toxoplasma gondii is a parasitic protozoan belonging to the phylum Apicomplexa, which contains many pathogenic species that are of great medical and veterinary importance, such as *Plasmodium* parasites that cause malaria and *Cryptosporidium* species that cause diarrhea. Pregnant women and individuals with compromised immune functions are at high risk to T. gondii infection because it often leads to abortion, stillbirth, or even death (1–4). In addition, T. gondii is also a big threat to livestock husbandry. Pigs and sheep are highly susceptible. In European countries, it was estimated that 11 to 23% of sheep abortions are caused by *Toxoplasma* infection (5). Like all other apicomplexan parasites, T. gondii has a complex life cycle that alternates among different developmental stages. The transmission between intermediate hosts is a unique property that distinguishes the life cycle of *Toxoplasma* from those of other apicomplexans (6). Tachyzoites during the acute infection phase and bradyzoites during chronic infection are the main forms of parasites in intermediate hosts. Tachyzoites actively invade host cells, replicate within them in a membrane-bound structure called parasitophorous vacuole (PV), and then egress from host cells when the PV grows big enough. The invasion-replication-egress steps form a lytic cycle of tachyzoites, and each cycle takes about 48 h. Within one lytic cycle, the parasites undergo 6 to 8 rounds of divisions through endodyogeny, giving rise to 64 or more parasites from one. At different phases of the lytic cycle and different stages of the cell cycle (G_1_, S, M, and C stages), the biological activities and gene expression of the parasites are quite different (7, 8).

*Toxoplasma* tachyzoites are able to infect all nucleated host cells, partially because they use a sophisticated and active invasion process to enter host cells (9). The invasion machinery is quite complex, consisting of proteins from both parasites and host cells (9, 10). Secretory proteins released from different organelles of *Toxoplasma* play key roles during invasion. Micronemal proteins MIC2 and AMA1 are required for close interaction with host cells, as well as for the formation of moving junction, a structure through which the parasite enters the host cell (11–14). Rhoptry proteins, particularly rhoptry neck proteins like RON2, RON4, RON5, and RON8, are also key for moving junction formation and therefore are critical for parasite invasion (13). These microneme and rhoptry proteins are stored in the secretory organelles called micronemes and rhoptries, respectively, which are unique organelles in apicomplexan parasites and have roles fundamental to the parasitic life style. During the initiation of invasion, micronemal proteins are released to the parasite’s surface from micronemes, whereas rhoptry neck proteins are secreted into host cells and form a complex at the cell periphery (10). AMA1 on parasite membrane and RON2 on host cell membrane interact with each other and form the key connection that drives moving junction establishment and facilitates parasite invasion (15–17). Invasion is a fast process and is completed within tens of seconds. This means that proteins involved in invasion are needed only during a very short period of the lytic cycle. Consistent with this, expression of many secretory proteins shows periodic changes during the cell cycle of the parasites (8, 18).

Over the past 20 years, numerous transcriptomic analyses have revealed the gene expression differences among different developmental stages or different phases of the lytic or cell cycle (19). Tight regulation leads to “just in time” expression of genes when their functions are needed. However, little is known about how such regulation is achieved. Historically, few transcription factors (TFs) have been identified in apicomplexan parasites, partially due to the low sequence similarity between parasite TFs and the TFs in model organisms. The paradox of sophisticated gene regulation and the paucity of recognizable TFs was partially solved with the discovery of ApiAP2 (apicomplexan AP2) factors in these parasites (20). These factors contain one or more AP2 domains that have sequence similarity to the Apetala2/ethylene response factor (AP2/ERF) integrase DNA-binding domain present in many plant TFs. AP2 domains are about 60 amino acids in length and form defined structures to bind DNA motifs in promoter regions of target genes to regulate their expression (21–23). AP2 factors are greatly expanded in apicomplexan parasites, with 27 in Plasmodium falciparum and 67 in T. gondii (24). Functional studies have shown that AP2 factors are indeed involved in gene expression regulation during parasite growth and development. A number of AP2s in *Plasmodium*, including AP2-G, AP2-O, and AP2-Sp, are found to play key roles for the progression of parasite life cycle. They regulate the expression of genes that control gametogenesis, ookinete maturation, oocysts formation, sporozoites production, and more (25–29). Similar findings have also been reported in *Toxoplasma*, but the majority of work has been focused on the function of AP2s during cell cycle progression and bradyzoite differentiation. Quite a few AP2s, including AP2IX-9, AP2IV-4, AP2IX-4, AP2IV-3, and AP2XI-4, suppress or activate the expression of specific genes during the transition from tachyzoites to bradyzoites (30–34). As such, they have a profound impact on the establishment of chronic information. In addition to binding to the promoter regions of target genes to directly regulate their expression, *Toxoplasma* AP2s may also interact with epigenetic factors to regulate gene expression. Four TgAP2s (IX-7, XI-2, X-8, and XII-4) were coimmunoprecipitated with the histone acetyltransferase GCN5b, which is crucial for parasite survival (35). At least 10 TgAP2s were found to interact with MORC, which recruits histone deacetylase HDAC3 to repress the expression of sexual-stage-specific genes at other stages (36). Therefore, TgAP2s also control the commitment to sexual development. In addition, TgAP2X-5 and TgAP2XI-5 were found to interact with each other and regulate the expression of genes that contribute to parasite virulence (37, 38). Most of the AP2s characterized so far do not play significant roles in tachyzoite growth *in vitro*. However, a recent genetic screen showed that a large number of AP2s do contribute to parasite fitness, the loss of which would result in reduced parasite growth (39). No AP2s of this type have been studied in detail so far. In this study, we found that TgAP2X-4 is such a factor. It regulates the expression of many cell cycle- and life cycle-regulated genes. In its absence, the parasites grow poorly *in vitro* and become avirulent in mice. This work further illustrated the function diversities of AP2s in apicomplexan parasites.

## RESULTS

### AP2X-4 is a Coccidia-specific AP2 factor localized to a parasite’s nucleus.

**FIG 1:**
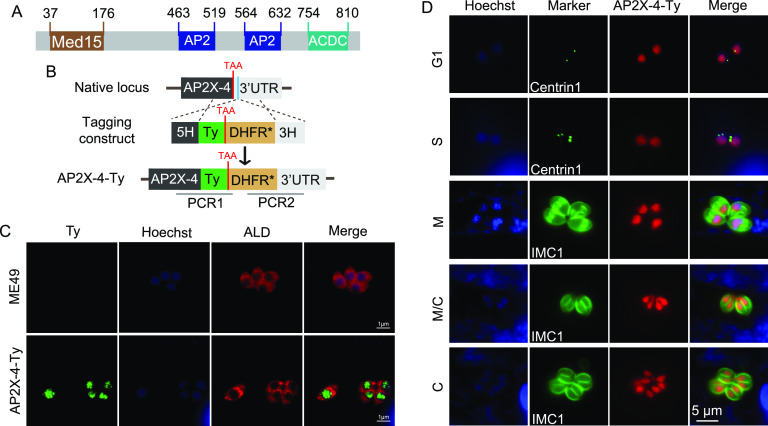
Domain structure and subcellular localization of TgAP2X-4. (A) Positions of the three recognizable domains in TgAP2X-4. ACDC, AP2-coincident domain mainly at the C terminus. (B) Diagram illustrating the insertion of a Ty tag to the endogenous locus of TgAP2X-4 in ME49, using CRISPR/Cas9-mediated homologous recombination. The blue bar indicates the CRISPR targeting site. PCR1 and PCR2 are used to identify the tagging clones. (C) Immunofluorescent staining of indicated strains using antibodies against Ty and TgALD. (D) Expression of AP2X-4 during the cell cycle of *Toxoplasma* tachyzoites, as determined by IFA examination of the ME49 AP2X-4-Ty strain.

The full-length AP2X-4 (TGME49_224050) protein from T. gondii contains 896 amino acids. Homology-based sequence analyses found four recognizable domains in TgAP2X-4, including a med15 domain at the N terminus (Fig. 1A), which is commonly found in the mediator complex and is an important molecular recognition site to facilitate the assembly of protein complexes involved in transcriptional regulation (40). It contains two AP2 domains in the center and one ACDC domain at the C terminus (Fig. 1A). The ACDC domain is present in a number of AP2 factors in apicomplexan parasites, but its function remains to be explored (41). A BLAST search indicated that AP2X-4 is present only in cyst-forming coccidian parasites such as *Neospora*, *Besnoitia*, and *Cyclospora*.

To examine the subcellular localization of TgAP2X-4 in parasites, a Ty epitope tag was fused to its C terminus at the endogenous gene locus (Fig. 1B). After diagnostic PCRs that identified the correct clones, immunofluorescence assays (IFA) were performed. In the transgenic parasites (AP2X-4-Ty), AP2X-4 was clearly localized to the parasite’s nucleus, as demonstrated by the colocalization of Ty staining signal with that of Hoechst (Fig. 1C). This is consistent with AP2X-4 being a putative transcription factor (20). The expression of a number of AP2 factors was reported to fluctuate during the cell cycle of *Toxoplasma* tachyzoites. To check whether the same occurred to TgAP2X-4, its expression at different stages (as indicated by IMC1 or Centrin1 costaining with AP2X-4-Ty) of the cell cycle was examined by IFA. The results showed that there was no obvious change across the cell cycle (Fig. 1D), suggesting that it is constitutively expressed in tachyzoites.

### AP2X-4 is critical for tachyzoites growth *in vitro*.

**FIG 2:**
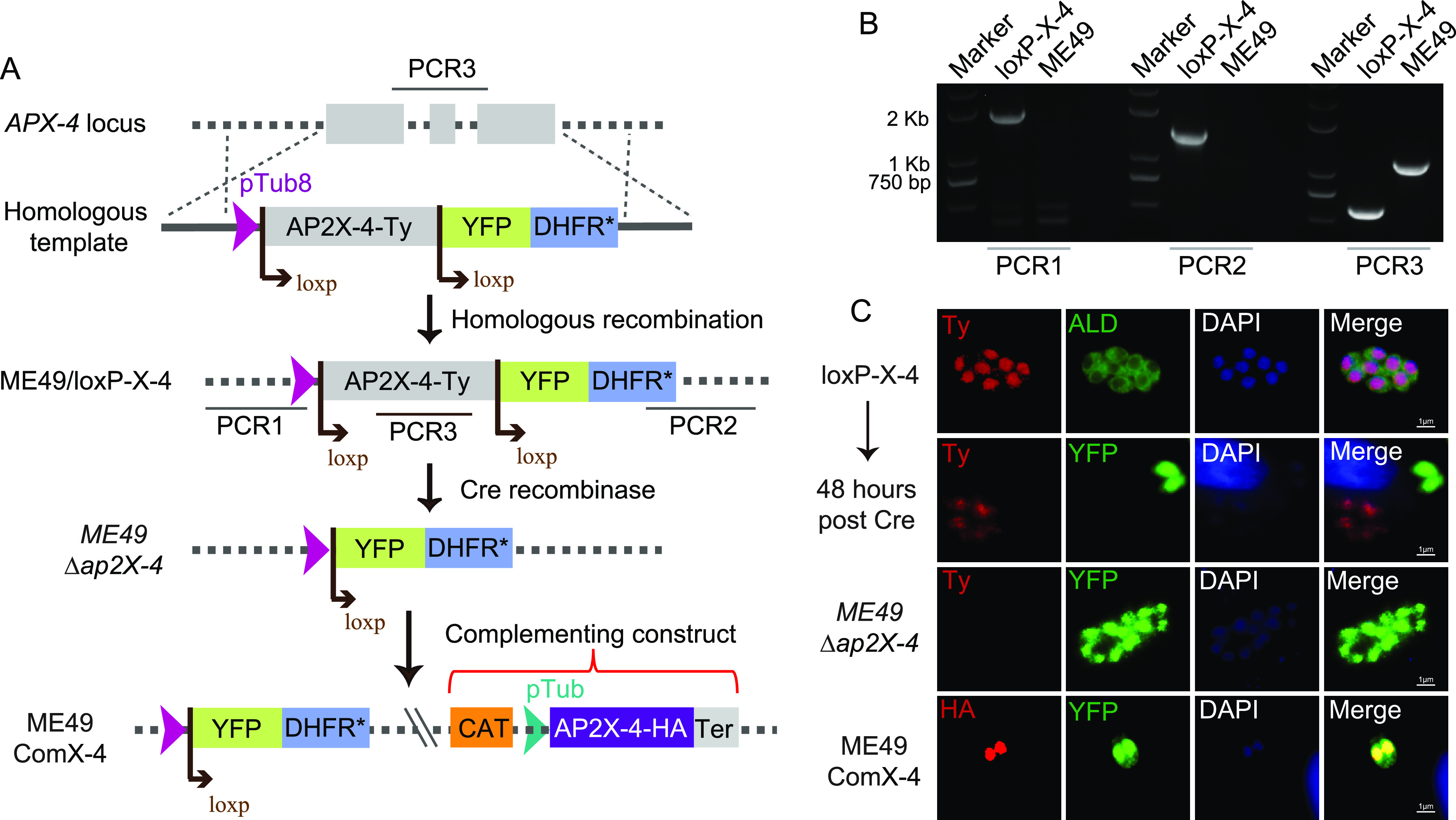
Construction of *TgAP2X-4* deletion and complementation strains. (A) Diagram illustration of the two steps used to construct the *TgAP2X-4* deletion strain ME49 *Δap2X-4*. First, a CRISPR/Cas9-assisted homologous recombination was used to generate the intermediate strain ME49/loxP-X-4, in which the endogenous *TgAP2X-4* was replaced with *AP2X-4* flanked by loxP sites. Then, a Cre recombinase-expressing plasmid was introduced into the ME49/loxP-X-4 strain to induce recombination between the two loxP sites, leading to the removal of *TgAP2X-4*. The complementation strain ME49 ComX-4 was constructed by inserting the complementing construct into the ME49 *Δap2X-4* mutant and selected with chloramphenicol. Ter, terminus of DHFR; CAT, chloramphenicol acetyltransferase. (B) Diagnostic PCRs on one ME49/loxP-X-4 clone. (C) IFA showing the expression of TgAP2X-4 in indicated strains. The sample “48 h post Cre” was a mixed population after transfecting the Cre-expressing plasmid into the ME49/loxP-X-4 strain. The rest are clonal strains.

In order to test the biological functions of TgAP2X-4, we tried to knock out the coding gene by CRISPR-Cas9-assisted homologous gene replacement in ME49. Despite multiple trials, we were not able to obtain single clones with TgAP2X-4 replaced by the selection marker DHFR*, which conferred pyrimethamine resistance to transgenic parasites. This indicated that TgAP2X-4 might have critical roles for tachyzoite growth, consistent with its low phenotype score (−3.71) in a genome-wide fitness screen (42). Alternatively, we used the loxP-Cre approach to modify the *TgAP2X-4* locus. First, the endogenous *TgAP2X-4* was replaced with the coding sequence of *TgAP2X-4* (tagged with Ty at the C terminus) that was flanked by two loxP sites, resulting in an intermediate strain called ME49/loxP-AP2X-4 (Fig. 2A). Then, a Cre recombinase-expressing plasmid was introduced into the ME49/loxP-AP2X-4 strain and selected for yellow fluorescent protein-positive (YFP^+^) parasites, which were TgAP2X-4 deletion mutants. Cre-mediated recombination between two loxP sites removed the *TgAP2X-4* gene and, meanwhile, brought the YFP-coding sequence to the Tub8 promoter, which then activated the expression of YFP. As such, YFP expression was an indication of TgAP2X-4 deletion. The ME49/loxP-AP2X-4 strain was readily obtained, as indicated by diagnostic PCR on single clones and nuclear localization of the Ty signal (Fig. 2B). After transfection of the Cre-expressing plasmid, a fraction of the parasites started to express YFP and lost the Ty signal (Fig. 2B and C). Single clones (ME49 Δ*ap2X-4*) that were YFP positive and TgAP2X-4 negative were isolated (Fig. 2B and C), suggesting that TgAP2X-4 was not essential for parasite survival, although it was indeed crucial for parasite growth (see below). To facilitate the subsequent phenotypic analyses, we also constructed a complementation strain (ME49/ComX-4) by reexpressing an HA-tagged TgAP2X-4 in the ME49 Δ*ap2X-4* mutant (Fig. 2B).

**FIG 3:**
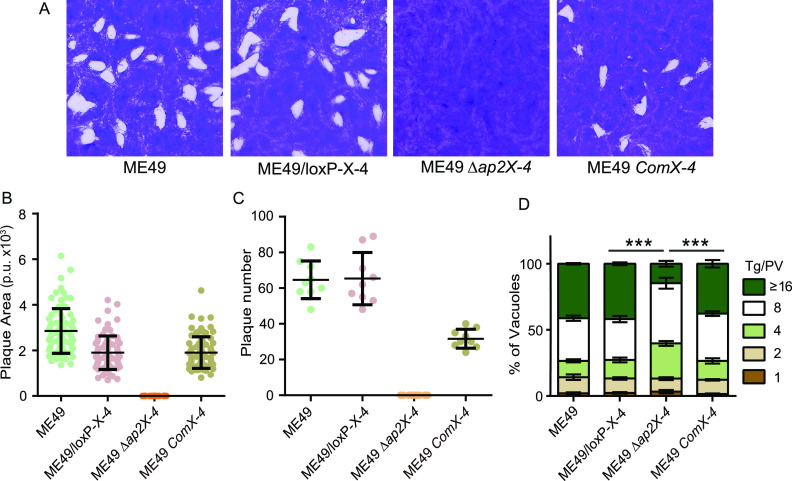
Impaired parasite growth caused by *TgAP2X-4* deletion. (A) A 10-day plaquing assay comparing the overall growth of indicated strains. (B) Sizes of plaques obtained from panel A, which were measured in Adobe Photoshop and expressed as pixel units. Over 80 plaques were analyzed for each strain. (C) Number of plaques in panel A; each strain was independently tested three times, each with triplicates. Means ± standard deviation (SD) were plotted. (D) Intracellular replication assay of indicated strains. Successfully invaded parasites were allowed to replicate for 36 h before determination of the number of parasites in each PV. Means ± standard error of the mean (SEM) of three independent experiments, ***, *P *< 0.001, two-way ANOVA.

To estimate the role of TgAP2X-4 during parasite growth, a 14-day plaque assay was performed. Although the ME49 Δ*ap2X-4* mutant was viable in tissue culture, it did not form any visible plaques in the 14-day plaque assay, whereas the parental strain ME49, loxP-AP2X-4, and the complement strain ComX-4 all formed big plaques (Fig. 3A to C). These data suggest a severe growth defect of the Δ*ap2X-4* mutant. We also did an intracellular replication assay to estimate the propagation rates of parasites after successful invasion. Similar to the plaque assay, the ME49 Δ*ap2X-4* mutant had a significant reduction in replication efficiency compared to that of AP2X-4-expressing parasites (Fig. 3D).

**FIG 4:**
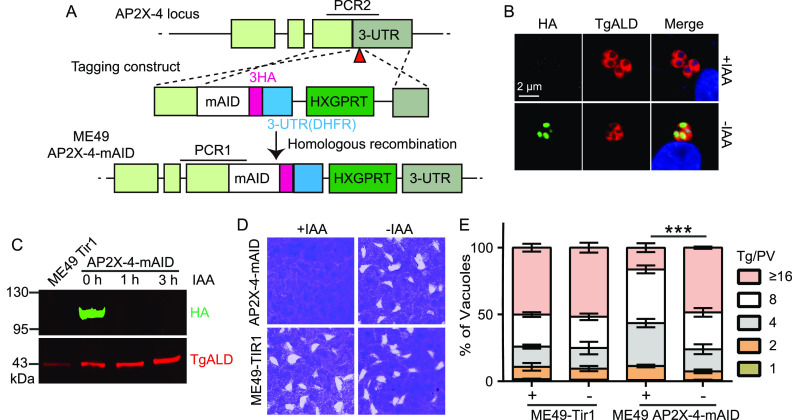
Estimation of the role of TgAP2X-4 in parasites through a conditional protein depletion approach. (A) Strategy used to insert an mAID domain to the C terminus of the endogenous TgAP2X-4. Red triangle indicates the CRISPR targeting site. PCR1 and PCR2 were used to screen single clones. (B) IFA analysis on a selected AP2X-4-mAID clone in the presence or absence of IAA, which induced the degradation of TgAP2X-4 as indicated by the HA staining signal. (C) Western blotting checking the degradation of AP2-X-4 by IAA treatment. The ME49 AP2X-4-mAID strain was treated with IAA for 0, 1, or 3 h and then probed with an anti-HA antibody. TgALD was included as a loading control. (D) Plaque assay showing the growth of strains with or without 500 μM IAA treatment. (E) Intracellular replication of the parental strain ME49-TIR1 or the ME49 AP2X-4-mAID strain in the presence or absence of IAA. Means ± SD of three independent experiments, ***, *P *< 0.001, two-way ANOVA.

Given the crucial role of AP2X-4 for parasite growth, we also tested its function using a conditional protein depletion approach. For this purpose, the endogenous AP2X-4 was tagged with an indole-3-acetic acid (IAA)-inducible degradation domain (mAID, which itself contained a triple HA tag) at the C terminus (Fig. 4A). Without IAA treatment, TgAP2X-4 was expressed and the parasites grew normally (Fig. 4B). Upon IAA induction, TgAP2X-4 was quickly degraded, as determined by the disappearance of HA 48 h after IAA treatment (Fig. 4B). Western blotting analyses suggest that AP2X-4 was degraded to undetectable levels within 1 h of IAA treatment (Fig. 4C). To assess the functional consequences of TgAP2X-4 depletion, plaque and intracellular replication assays were performed in the presence or absence of IAA. Similar to the ME49 Δ*ap2X-4* mutant, conditional depletion of TgAP2X-4 also blocked plaque formation and reduced parasite proliferation (Fig. 4D and E). Therefore, both gene deletion and protein depletion results pointed out a critical role of TgAP2X-4 in the lytic cycle of tachyzoites.

To further examine the role of TgAP2X-4 in other strains, we constructed a TgAP2X-4 deletion mutant in the type I strain RH-DiCre (43), which expressed a split Cre recombinase that could be reconstituted by rapamycin. The intermediate strain DiCre/loxP-AP2X-4 was constructed in a way similar to that described above for ME49/loxP-AP2X-4. Then, rapamycin was added and YFP-expressing *TgAP2X-4* deletion parasites were selected and verified. Both plaque and intracellular assays demonstrated that the growth of the *TgAP2X-4* deletion mutant was greatly impaired, similar to what was observed in the type II strain ME49 (Fig. S1A to D). In addition, the RH Δ*ap2X-4* mutant also displayed significantly reduced invasion efficiency and virulence, which could be fully restored by AP2X-4 complementation (Fig. S1E and F). Together, these results suggest that AP2X-4 is critical for *Toxoplasma* growth in diverse strains, although it is not absolutely essential.

### AP2X-4 controls the expression of cell cycle-regulated genes and is required for efficient invasion into host cells.

**FIG 5:**
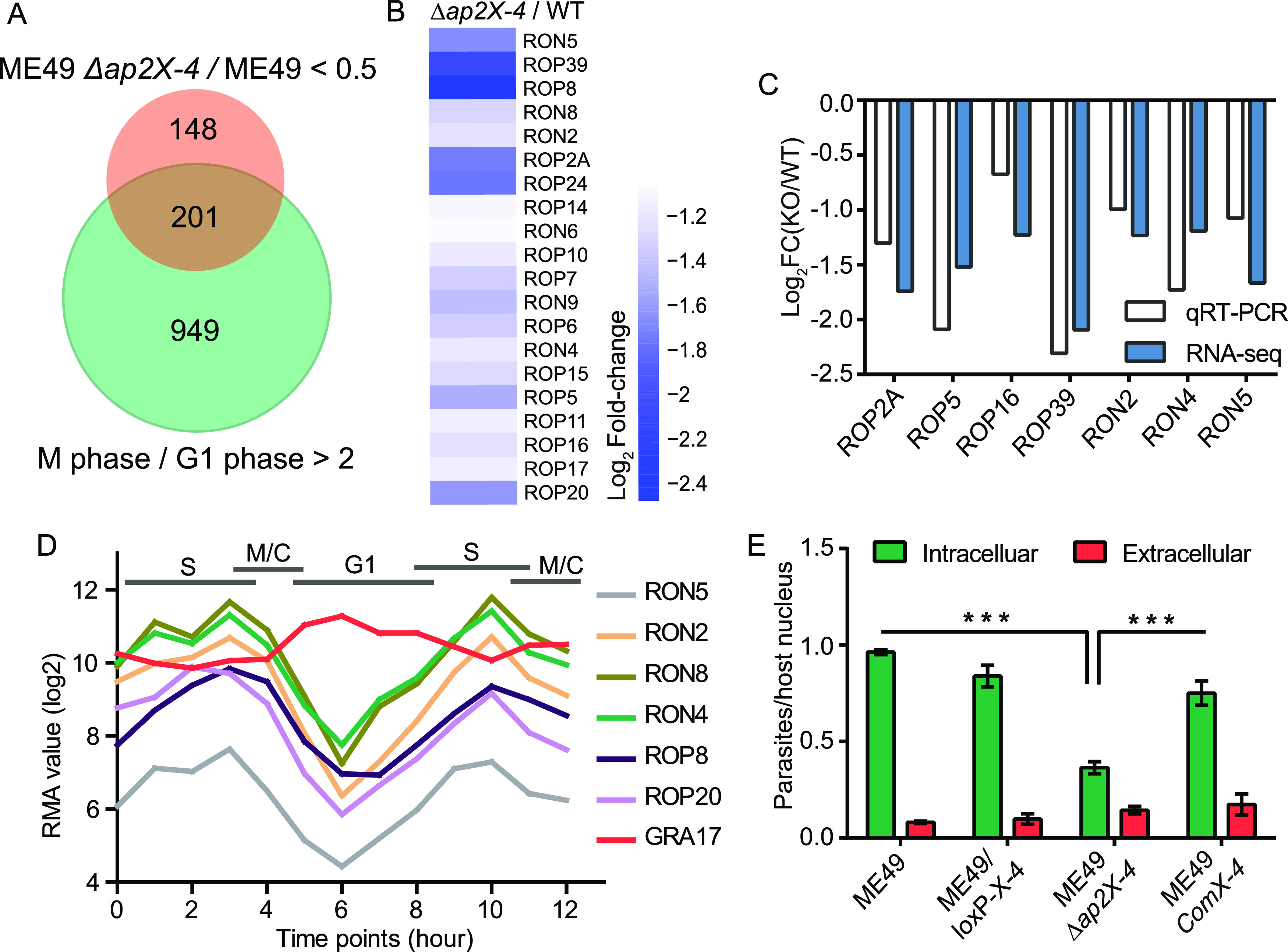
*TgAP2X-4* disruption leads to altered expression of cell cycle-regulated genes and causes reduced host cell invasion. (A) Venn diagram showing the overlap of genes whose expressions are upregulated during the M phase (green) and those whose expressions are decreased upon *TgAP2X-4* deletion (orange). Data for gene expression changes during the cell cycle were from ToxoDB (data set of Cell Cycle Expression Profiles [RH] [7]), and those for TgAP2X-4 deletion were obtained by RNA-Seq in this study. (B) Rhoptry genes with significantly reduced expression after AP2X-4 inactivation, as determined by RNA-Seq. (C) Expression changes of selected rhoptry genes in tachyzoites of ME49 after AP2X-4 deletion were verified by RNA-Seq. (D) Transcription of many rhoptry genes is cell cycle regulated. Data from ToxoDB, GRA17 is included as a control. (E) Efficiencies of host cell invasion by tachyzoites of indicated strains were determined by a two-color assay that distinguishes intracellular and extracellular parasites. Mean ± SEM of three independent experiments, ***, *P *< 0.001, one-way ANOVA with Bonferroni’s post test.

To understand the molecular mechanisms underlying the poor growth of the Δ*ap2X-4* mutant, we sought to estimate the genes that might be regulated by AP2X-4. First, we tried chromatin immunoprecipitation sequencing (ChIP-Seq) to assess the binding sites of AP2X-4 in the parasite genome as a way to infer the direct targets of AP2X-4. Both the AP2X-4-Ty and the AP2X-4-mAID strains (using Ty and HA antibodies for precipitation, respectively) were used for this purpose. Unfortunately, these experiments were not successful since very few specific DNA fragments could be enriched in the experimental group compared to those in the control group (using mouse IgG for precipitation). The exact reason for this lack of DNA enrichment is currently unknown. Alternatively, RNA-Seq analysis was used to determine the gene expression changes upon TgAP2X-4 inactivation in ME49. The results showed that, consistent with TgAP2X-4 being a putative transcription factor, its deletion resulted in expression changes of 1,479 genes (Table S2), among which 349 genes (besides TgAP2X-4 itself) were significantly downregulated (transcript level decreased more than 2-fold; *P *< 0.05). A close look at the downregulated genes found that over 200 (~57%) of them were cell cycle-regulated in wild-type tachyzoites (Fig. 5A), with significant mRNA abundance differences between the M phase and G_1_ phase. Moreover, among the downregulated genes, there are more than 20 rhoptry protein-encoding genes, including rhoptry neck proteins such as RON2, RON5, and RON8, as well as canonical rhoptry proteins like ROP2A (Fig. 5B). Expression changes of these rhoptry proteins was further confirmed by real-time PCR (RT-PCR) (Fig. 5C), which reported a 2- to 4-fold reduction for the mRNA levels of these rhoptry proteins after *TgAP2X-4* deletion. RT-PCR also found downregulation of rhoptry proteins in the RH Δ*ap2X-4* mutant, which was restored by *AP2X-4* complementation (Fig. S2). Interestingly, all these rhoptry proteins have very similar periodic expression pattern during the cell cycle, which peaked at late S and early M/C phases and bottomed at the G_1_ phase (Fig. 5D).

Among the rhoptry proteins whose expressions are influenced by TgAP2X-4, rhoptry neck proteins like RON2, RON5, and RON8 play crucial roles during parasite invasion, as they are required for the establishment and proper function of the moving junction. As such, we sought to determine the impact of *TgAP2X-4* deletion on the efficiency of host cell invasion. The results showed that the invasion efficiency decreased 60% upon *TgAP2X-4* deletion (Fig. 5E), which was fully restored by *TgAP2X-4* complementation. Together, these results suggest that inactivation of *TgAP2X-4* causes reduced expression of rhoptry neck proteins, which in turn resulted in decreased invasion.

### AP2X-4 affects the expression of many developmentally regulated genes.

**FIG 6:**
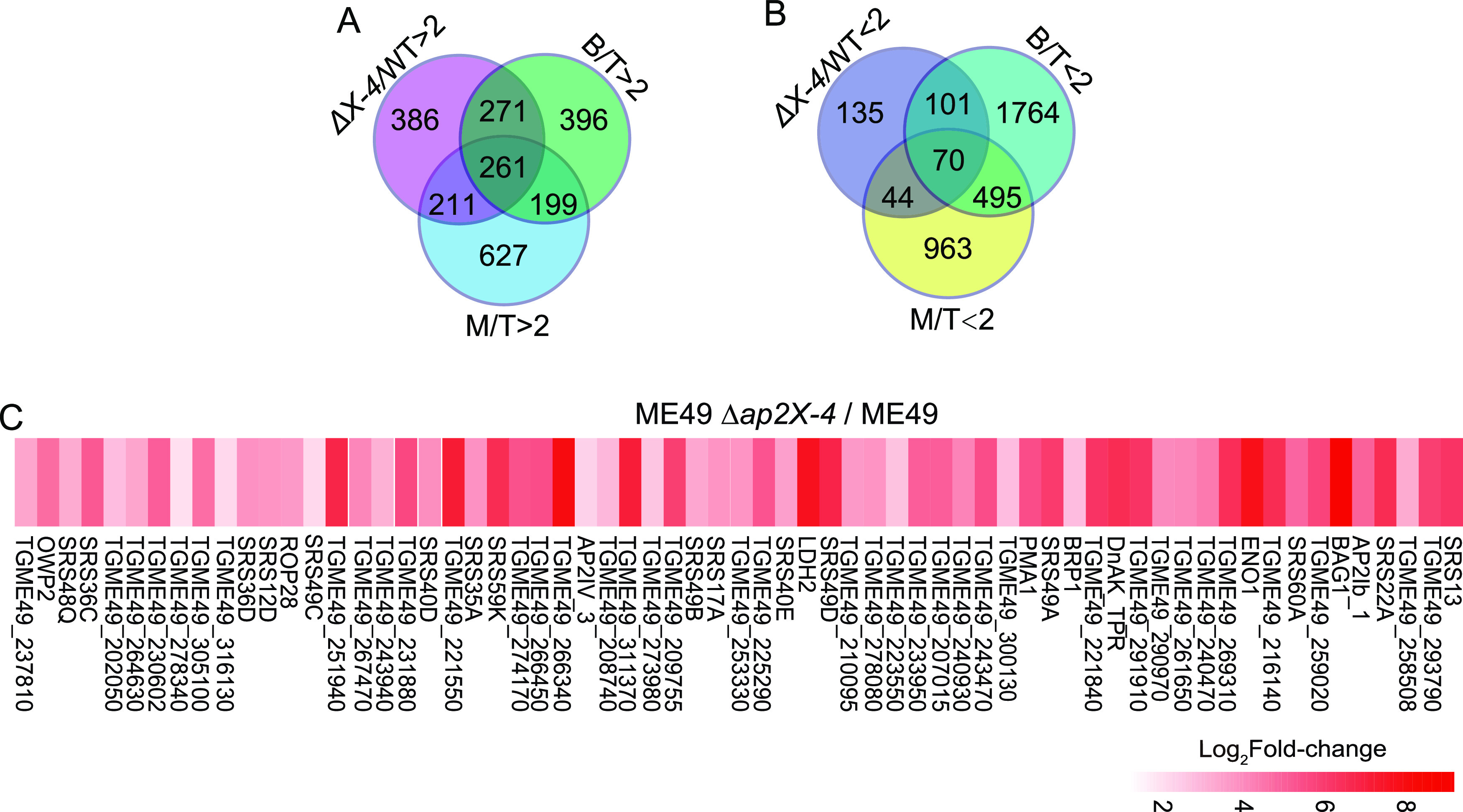
*TgAP2X-4* deletion causes increased expression of genes that are normally upregulated in bradyzoites and merozoites in the tachyzoite stage. (A and B) Venn diagram showing the overlap of genes whose expressions are upregulated (A) or downregulated (B) in bradyzoites (B/T of >2 or <2, data from the ToxoDB data set Feline Enterocyte, Tachyzoite, Bradyzoite Stage Transcriptome [Hehl, Ramakrishnan et al.]) or merozoites (M/T of >2 or <2, data from the ToxoDB data set Tachyzoite and merozoite transcriptomes [Hehl et al.]) and those whose expressions are upregulated upon TgAP2X-4 deletion (ΔX-4/WT of >2 or <2, obtained from RNA-Seq in this study). (C) Heat map showing the increased expression of selected bradyzoite genes in tachyzoites of the ME49 Δ*ap2X-4* mutant. RNA-Seq data were used to generate this graph.

RNA-Seq analyses revealed over 1,000 genes whose expression was significantly increased upon *AP2X-4* deletion. Among the upregulated genes, 743 genes (~65.8%) are developmentally regulated, with higher expression in bradyzoites or merozoites than in tachyzoites in wild-type strains (Fig. 6A). Similarly, among the 350 genes that are downregulated after *AP2X-4* disruption, 215 (~61.4%) are developmentally regulated, with lower expression in bradyzoites or merozoites than in tachyzoites (Fig. 6B). A previous transcriptomic study compared the gene expression differences between ME49 tachyzoites and alkaline (pH = 8.2) induced bradyzoites and identified 168 genes whose expression increased more than 5-fold upon bradyzoite induction (ToxoDB) (44). Of those 168 genes, 105 were upregulated (>5-fold) in tachyzoites of the Δ*ap2X-4* mutant compared with those of ME49. Among these genes, BAG1 (594.5-fold), ENO1 (223.5-fold), and LDH2 (203.6-fold) had the most dramatic increase after *AP2X-4* inactivation (Fig. 6C). These results demonstrate that AP2X-4 is an important factor directly or indirectly involved in the control of proper expression of bradyzoite and merozoite genes in tachyzoites.

### The virulence of AP2X-4 deficient mutants is drastically attenuated *in vivo*.

**FIG 7:**
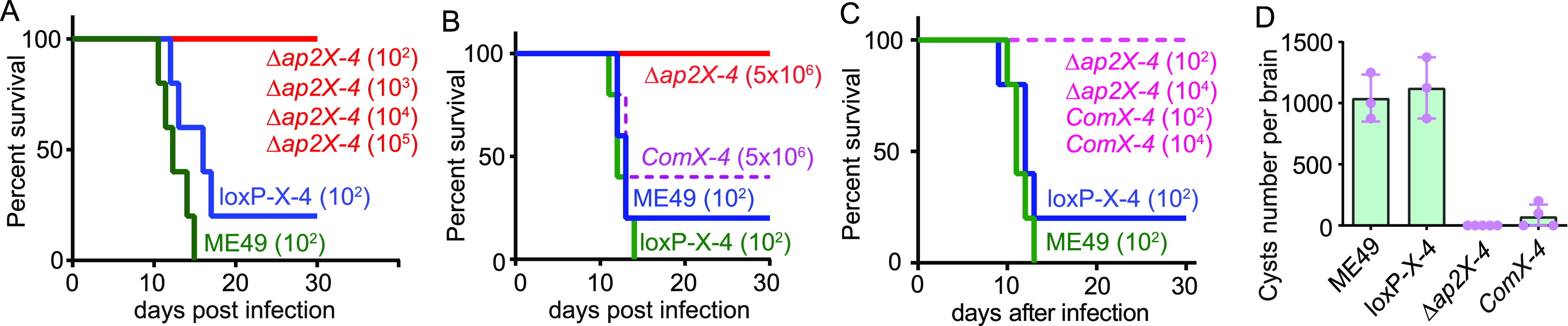
The virulence of the *AP2X-4* deletion mutant is significantly attenuated *in vivo*. (A to C) Survival of ICR mice infected with tachyzoites of indicated strains, 10^2^ to 10^6^ denote the infection dose per mouse. Each strain or infection dose was tested with five mice. (D) Number of *Toxoplasma* cysts in the brains of mice that survived the infection. Thirty days postinfection, tissue cysts in mouse brains were examined by DBA-FITC staining and counted. Mean ± SD of *n* = 3 or more mice for each strain.

Given the complex gene regulation set by AP2X-4, as well as the crucial role of AP2X-4 for tachyzoite growth *in vitro*, we wanted to test whether AP2X-4 has any role *in vivo* during parasite infection. ICR mice were infected with *AP2X-4*-expressing or *AP2X-4*-deleted parasites at different doses through intraperitoneal injection. Then, the symptoms and survival of infected mice were monitored daily. Consistent with the growth defects *in vitro*, ME49 Δ*ap2X-4* was severely attenuated in mice (Fig. 7A). At an infection dose of 100 tachyzoites per mouse, the parental strain ME49 killed almost all mice within 16 days. However, for ME49 Δ*ap2X-4*, even at the dose of 5 × 10^6^ per mouse, it did not cause apparent symptoms or animal death, suggesting a significant attenuation of virulence (Fig. 7A and B). The loss of virulence in the ME49 Δ*ap2X-4* mutants was partially rescued by AP2X-4 complementation (Fig. 7B), because at high infection dose (5 × 10^6^ per mouse), the ComX-4 strain caused 60% mortality whereas the Δ*ap2X-4* did not. It should be mentioned that at a low infection dose of 10^2^ or 10^4^ per mouse, ComX-4 did not cause mouse death as ME49 or the LoxP-X-4 strains did (Fig. 7C), suggesting a lack of full rescue in virulence, which is likely because the complementing AP2X-4 was driven by the tubulin-α1 promoter instead of its own promoter, which made it hard to achieve the wild-type level of protein expression, as well as transcriptional regulation. Nonetheless, a nearly full rescue of growth defects *in vitro* and partial rescue of virulence in mice after complementation suggest that the defects are highly likely to be caused by *AP2X-4* inactivation. Since *AP2X-4* deletion affected the expression of many bradyzoite specific genes, we also tested the cyst formation in mice infected with ME49 Δ*ap2X-4*. As a reference, ME49 and the LoxP-X-4 strain produced about 1,000 cysts per mouse brain 30 days after infection. However, no cyst was found in mice infected with ME49 Δ*ap2X-4*, and AP2X-4 complementation partially restored this cyst formation defect (Fig. 7D). These results suggest that AP2X-4 is also critical for the establishment of chronic infection.

## DISCUSSION

T. gondii has sophisticated strategies to regulate its gene expression to cope with its complex life style. However, the underlying regulatory mechanisms are largely unknown. Here, we identified and characterized an AP2 family transcription factor, AP2X-4. Genetic analyses showed that AP2X-4 is crucial for tachyzoite growth. Mutants lacking AP2X-4 displayed altered expression of a large number of cell cycle- and life cycle-regulated genes, leading to reduced host cell invasion and intracellular replication *in vitro*, as well as attenuated virulence *in vivo*. This mode of action of AP2X-4 is different from those of many other characterized AP2 factors in *Toxoplasma* that regulate bradyzoite differentiation, which are typically dispensable for tachyzoite growth and survival. A recent genetic screen found that many ApiAP2 factors were involved in the fitness of tachyzoites (39). As such, dissection of the function and working mechanisms of AP2X-4 has important insights into the study of other ApiAP2 factors that are implicated in tachyzoite growth and fitness.

RNA-Seq analysis found that AP2X-4 directly or indirectly regulates the expression of life cycle- and cell cycle-regulated genes. The expression of a large number of genes that are normally induced or specifically expressed in bradyzoites was upregulated in tachyzoites of the Δ*ap2X-4* mutant. These include the bradyzoite markers BAG1, LDH2, and ENO1. In this regard, AP2X-4 serves as a transcription repressor to suppress the expression of bradyzoite genes in tachyzoites, which is similar to a previously reported AP2 factor, AP2IV-4 (32). Deletion of *AP2IV-4* did not affect tachyzoite growth, but it resulted in mistiming of bradyzoite gene expression in tachyzoites. As a consequence, it led to virulence attenuation in the type II strain PRU and loss of tissue cyst formation in mice, which was thought to be caused by an overly activated inflammatory immune response that eliminated the parasites during acute infection (32). Given the similar regulation of bradyzoite genes by AP2X-4, improper activation of host immune response by misregulated bradyzoite antigens could also be an important contributing factor to the reduced virulence of the Δ*ap2X-4* mutant, in addition to its poor growth and invasion defect. In addition, *AP2X-4* deletion led to decreased expression of many rhoptry proteins. A previous gene knockout screen targeting 27 ROP gene loci identified 17 that affected cyst formation *in vivo* (45). Of these 17 loci, ROP5, ROP17, ROP7, ROP2, ROP8, and ROP16 were regulated by AP2X-4 (Fig. 5C). Therefore, AP2X-4 may affect parasite activity *in vivo* through these ROP proteins.

In addition, the expression of a large number of cell cycle-regulated genes is also influenced by AP2X-4. Among these genes are those that encode a subset of RON proteins that displayed decreased expression after AP2X-4 deletion. As a consequence, the efficiency of host cell invasion was severely impaired in the Δ*ap2X-4* mutant, leading to the poor growth of the mutant *in vitro*. However, the expression of AP2X-4 itself does not seem to fluctuate much during the cell cycle, as indicated by the mRNA (data from ToxoDB) and protein levels (Fig. 1D). This suggests that additional factors may be involved to ensure the timely expression of cell cycle-regulated genes along with AP2X-4.

AP2X-4 is involved in the regulation of multiple genes, but the exact mechanisms are currently unknown. Accumulating evidence suggests that ApiAP2 factors may interact with each other or with epigenetic factors such as MORC, HDAC3, and GCN5b to achieve desired gene regulation (35, 36). In this sense, it is possible that AP2X-4 interacts with different partners to regulate different target genes. Interestingly, the expression of 16 AP2 factors was altered in the Δ*ap2X-4* mutant, which includes AP2X-5 (Table S2). Previous work has demonstrated that AP2X-5 and AP2XI-5 form a complex to regulate the expression of virulence genes that include those that encode rhoptry proteins (38). Some of the ROPs (such as ROP15 and ROP24) targeted by AP2X-5 and AP2XI-5 are also regulated by AP2X-4. Thus, it is possible that there is some level of cross talk between AP2X-4 and AP2X-5/AP2XI-5 to ensure precise regulation of these rhoptry proteins. As such, a full dissection of the AP2X-4 interaction network will be important to understand its exact working mechanisms, which deserve further investigations.

## MATERIALS AND METHODS

### Parasites and culture conditions.

The type II strain ME49 and type I strain RH-DiCre (RH Δ*hxgprt* Δ*ku80*/DiCre-CAT; provided by Moritz Treeck from the Francis Crick Institute, United Kingdom) (46) were used in this study. ME49-TIR1 was constructed by introducing the pTUB1: OsTIR1-3FLAG~CAT fragment (amplified from Addgene plasmid no. 87258) into ME49 tachyzoites, as described previously (47). ME49 Δ*ap2X-4*, RH-DiCre Δ*ap2X-4*, their complementation strains, and ME49-TIR1/AP2X-4-mAID were constructed as described below. All parasites were propagated in human foreskin fibroblast (HFF) cells, which were cultured in Dulbecco’s modified Eagle medium supplemented with 10% fetal bovine serum, 100 unit/mL penicillin, and 100 μg/mL streptomycin. IAA at a final concentration of 500 μM was used to induce AP2X-4 degradation in the ME49-TIR1/AP2X-4-mAID strain, and rapamycin at a final concentration of 5 μM was used to induce AP2X-4 excision in the RH-DiCre/loxP-AP2X-4, when indicated.

### Endogenous tagging of AP2X-4 with the Ty tag.

CRISPR-Cas9-mediated homologous recombination was used to insert a Ty epitope tag to the C terminus of endogenous AP2X-4. A CRISPR plasmid targeting the 3′ end (close to the stop codon) of AP2X-4 was constructed using previously established methods (48). Primers used are listed in Table S1. The tagging construct consists of a Ty tag and the selection marker DHFR*, which were flanked by two homology arms (5H and 3H). The Ty tag and DHFR* were amplified from pUC19-Ty-3′-UTR-DHFR* as a single PCR product. The two homology arms were amplified from genomic DNA of ME49. Purified amplicons were ligated into pUC19 by the ClonExpress MultiS one-step cloning kit (Vazyme, Nanjing, China). Then, the CRISPR plasmid and tagging construct were cotransfected into purified ME49 tachyzoites and selected with 1 μM pyrimethamine. Tagged lines were examined by diagnostic PCRs to confirm correct integration before immunofluorescent analyses.

### Construction of the AP2X-4 deletion and complementation strains.

The plasmid pAP2X4::DHFR, which contained the 5′ untranslated region (5′-UTR) and 3′-UTR (about 1 kb in size) of AP2X-4 as homologous arms, as well as DHFR* as the selection marker, was used to directly knock out AP2X-4. The ME49/loxP-AP2X-4 strain was constructed by replacing endogenous AP2X-4 with a floxing construct (loxP-AP2X-4-Ty-LoxP-YFP-DHFR*), which was generated by cloning the following fragments into pAP2X4::DHFR to replace DHFR*: the coding sequence of AP2X-4 amplified from ME49 cDNA, the Tub8 promoter-loxP fragment and the Ty-loxP-YFP-DHFR* fragment amplified from pLoxP-KillerRed-YFP~DHFR*, a modified version of p5RT70loxPKillerRedloxPYFP–HX (43) (primers listed in Table S1). The floxing strain ME49/loxP-AP2X-4 was constructed in a way similar to that of the Ty-tagging strain described above. Subsequently, the Cre recombinase-expressing plasmid pmin-Cre-eGFP was transfected into loxP-AP2X-4, and YFP^+^ parasites were selected (49). Single clones (YFP^+^) of ME49 Δ*ap2X-4* were then isolated, and loss of AP2X-4 was confirmed by PCR and IFA (anti-Ty). To construct the complementing strain ME49 Com*X-4*, a PCR amplicon containing pTub:AP2X-4-HA~CAT was transfected into ME49 Δ*ap2X-4* tachyzoites and selected with 30 μM chloramphenicol. Clonal strains of ME49 Com*X-4* were verified by IFA (anti-hemagglutinin [anti-HA]) to confirm the expression of AP2X-4. The RH-DiCre Δ*ap2X-4* and RH-DiCre Com*X-4* strains were constructed in a similar way, except that RH-DiCre was used as the parental strain and the excision of AP2X-4 was induced by rapamycin.

### Generation of the ME49-TIR1/AP2X-4-mAID strain.

Similar to Ty tagging, the AP2X-4-mAID strain was constructed by fusing an mAID construct to the C terminus of endogenous AP2X-4. The mAID construct contained mAID-3HA-3′-UTR(DHFR)~HXGPRT flanked by 5′ and 3′ homologous arms. It was generated as follows. The mAID-3HA-3′-UTR(DHFR)~HXGPRT fragment was amplified from pTUB1:YFP-mAID-3HA (Addgene plasmid no. 87259) as a single PCR product. The 5′ and 3′ homologous arms were amplified from genomic DNA of ME49 using primers listed in Table S1. Subsequently, these fragments were cloned into pUC19 by a one-step cloning kit (Vazyme, Nanjing, China). To construct the ME49-TIR1/AP2X-4-mAID strain, the CRISPR plasmid targeting the 3′ end of AP2X-4 was cotransfected with the PCR amplicon containing the mAID construct into ME49/TIR1 and selected with 25 μg/mL mycophenolic acid and 25 μg/mL xanthine (Sigma-Aldrich, St. Louis, MO, USA). Single clones were screened by diagnostic PCRs. IAA at a final concentration of 500 μM was used to induce AP2X-4 degradation when needed (47).

### Immunofluorescence assays.

Parasite-infected HFF cells were fixed with 4% paraformaldehyde for 15 min and then subjected to immunostaining following previously described protocols (50). Primary antibodies used include mouse anti-Ty monoclonal antibody, rabbit anti-TgALD polyclonal antibody, rabbit anti-TgIMC1 polyclonal antibody, mouse anti-HA monoclonal antibody (Medical & Biological Laboratories Co., Beijing, China), and rabbit anti-YFP polyclonal antibody (Proteintech, Chicago, USA). Primary antibodies were detected by Alexa-488- or Alexa-594-conjugated secondary antibodies (Life Technologies, CA, USA). Samples were visualized under the Olympus BX53 microscope (Olympus Life Science, Tokyo, Japan) equipped with an AxioCam 503 mono camera (Zeiss, Oberkochen, Germany).

### Tachyzoite growth and replication assays.

The overall fitness and growth of tachyzoites were estimated by plaque assays in HFF seeded 6-well plates, as described previously (50). The efficiencies of intracellular propagation after successful invasion were determined by replication assays, which allowed invaded parasites to replicate for 36 h before the number of parasites in each vacuole were counted. For both plaque and replication assays, each strain was tested at least three times independently.

### Invasion assay.

A classic two-color assay was used to estimate the invasion efficiency of different strains (51). Briefly, freshly egressed tachyzoites were allowed to invade HFF monolayer seeded on coverslips (10^6^ tachyzoites per well) at 37°C for 1 h. Then, the slides were washed three times to remove noninvaded parasites, fixed, and blocked for immunostaining. Extracellular parasites were stained with mouse anti-SAG1 antibody before permeabilization, whereas intracellular parasites were stained with rabbit anti-ALD antibody after Triton X-100 permeabilization. Alexa-conjugated secondary antibodies were used to visualize SAG1^+^ or ALD^+^ parasites. Parasites that were SAG1^+^ and ALD^+^ were attached but failed to invade. SAG1^−^ and ALD^+^ parasites were those that successfully invaded. Invasion efficiency was expressed as the number of invaded parasites per host nucleus.

### Induced egress assay.

Parasites were cultured in HFF monolayers seeded on coverslips in 24-well plates for 32 h. Then, the cultures were washed with prewarmed phosphate-buffered saline (PBS) and treated with 2 μM calcium ionophore A23187 at 37°C for 2 min (or 1% dimethyl sulfoxide [DMSO] as the control). Subsequently, the samples were fixed and stained with mouse anti-SAG1 antibody after permeabilization with 0.1% Triton X-100 (Sigma-Aldrich, St. Louis, MO, USA). Alexa 488-conjugated goat anti-mouse IgG was used as a secondary antibody to visualize the integrity of parasitophorous vacuoles (PVs). The number of both egressed and intact PVs was counted, and the egress efficiency was calculated by comparing the number of egressed PVs to that of total PVs.

### RNA-seq and real-time PCR to compare gene expression levels.

Freshly egressed tachyzoites were collected and purified through polycarbonate membranes with a pore size of 3 μm. Samples were washed with PBS, and total RNA was extracted using the TRIzol methods according to the manufacturer’s instructions (TransGen Biotech, Beijing, China). Three samples were independently prepared for each strain. Then, RNA libraries were constructed using the TruSeq RNA sample preparation kit (Illumina, San Diego, CA, USA) and sequenced with the Illumina HiSeq X Ten sequencer.

The raw sequencing reads were trimmed and quality controlled by SeqPrep and Sickle. Then, clean reads were mapped to the reference genome ME49 (downloaded from ToxoDB) using TopHat. To identify differentially expressed genes, the transcript level for each gene was calculated according to the fragments per kilobase of exon per million mapped reads (FPKM) method and then compared.

To validate the gene expression changes revealed by RNA-Seq, RNA samples were also subjected to quantitative RT-PCR analysis using the Fast SYBR method (Vazyme Biotech Co., Nanjing, China). Primers used are listed in Table S1. Expression of target genes was normalized to that of β-tubulin and then compared. Each sample was independently analyzed three times, each in triplicate.

### Virulence assay and cyst counting.

Seven-week-old female ICR mice were infected with freshly egressed tachyzoites by intraperitoneal injection. Each strain or infection dose was tested by 5 mice. Symptoms and survival of infected mice were monitored daily for 30 days. At the conclusion of the virulence test, mice that survived the infections were euthanized and the number of *Toxoplasma* cysts in the brains was determined by Dolichos biflorus agglutinin-fluorescein isothiocyanate (DBA-FITC; Vector Laboratories, Burlingame, CA, USA) staining. All animal work was approved by the Ethical Committee of Huazhong Agricultural University (permit no. HZAUMO-2019-039).

### Statistical analyses.

All statistical analyses were performed in GraphPad Prism (GraphPad Software, La Jolla, CA, USA), using Student’s *t* test, one-way analysis of variance (ANOVA), or two-way ANOVA, as indicated in figure legends.

### Data availability.

The RNA-Seq data generated in this study have been deposited to the Gene Expression Omnibus (GEO) database with the accession number GSE193779.

## References

[1] Luft BJ, Remington JS. 1992. Toxoplasmic encephalitis in AIDS. Clin Infect Dis 15:211–222. doi:10.1093/clinids/15.2.211. 1520757

[2] Montoya JG, Liesenfeld O. 2004. Toxoplasmosis. Lancet 363:1965–1976. doi:10.1016/S0140-6736(04)16412-X. 15194258

[3] Shojaee S, Teimouri A, Keshavarz H, Azami SJ, Nouri S. 2018. The relation of secondary sex ratio and miscarriage history with Toxoplasma gondii infection. BMC Infect Dis 18:307. doi:10.1186/s12879-018-3228-0. 29976155 PMC6034284

[4] Villena I, Ancelle T, Delmas C, Garcia P, Brezin AP, Thulliez P, Wallon M, King L, Goulet V. 2010. Congenital toxoplasmosis in France in 2007: first results from a national surveillance system. Euro Surveill 15:19600. doi:10.2807/ese.15.25.19600-en. 20587361

[5] Dubey JP. 2009. Toxoplasmosis in sheep–the last 20 years. Vet Parasitol 163:1–14. doi:10.1016/j.vetpar.2009.02.026. 19395175

[6] Dubey JP. 2008. The history of Toxoplasma gondii–the first 100 years. J Eukaryot Microbiol 55:467–475. doi:10.1111/j.1550-7408.2008.00345.x. 19120791

[7] Behnke MS, Wootton JC, Lehmann MM, Radke JB, Lucas O, Nawas J, Sibley LD, White MW. 2010. Coordinated progression through two subtranscriptomes underlies the tachyzoite cycle of Toxoplasma gondii. PLoS One 5:e12354. doi:10.1371/journal.pone.0012354. 20865045 PMC2928733

[8] Gaji RY, Behnke MS, Lehmann MM, White MW, Carruthers VB. 2011. Cell cycle-dependent, intercellular transmission of Toxoplasma gondii is accompanied by marked changes in parasite gene expression. Mol Microbiol 79:192–204. doi:10.1111/j.1365-2958.2010.07441.x. 21166903 PMC3075969

[9] Frenal K, Dubremetz JF, Lebrun M, Soldati-Favre D. 2017. Gliding motility powers invasion and egress in Apicomplexa. Nat Rev Microbiol 15:645–660. doi:10.1038/nrmicro.2017.86. 28867819

[10] Shen B, Sibley LD. 2012. The moving junction, a key portal to host cell invasion by apicomplexan parasites. Curr Opin Microbiol 15:449–455. doi:10.1016/j.mib.2012.02.007. 22445360 PMC3387501

[11] Carruthers VB, Giddings OK, Sibley LD. 1999. Secretion of micronemal proteins is associated with toxoplasma invasion of host cells. Cell Microbiol 1:225–235. doi:10.1046/j.1462-5822.1999.00023.x. 11207555

[12] Mordue DG, Desai N, Dustin M, Sibley LD. 1999. Invasion by Toxoplasma gondii establishes a moving junction that selectively excludes host cell plasma membrane proteins on the basis of their membrane anchoring. J Exp Med 190:1783–1792. doi:10.1084/jem.190.12.1783. 10601353 PMC2195726

[13] Alexander DL, Mital J, Ward GE, Bradley P, Boothroyd JC. 2005. Identification of the moving junction complex of Toxoplasma gondii: a collaboration between distinct secretory organelles. PLoS Pathog 1:e17. doi:10.1371/journal.ppat.0010017. 16244709 PMC1262624

[14] Mital J, Meissner M, Soldati D, Ward GE. 2005. Conditional expression of Toxoplasma gondii apical membrane antigen-1 (TgAMA1) demonstrates that TgAMA1 plays a critical role in host cell invasion. Mol Biol Cell 16:4341–4349. doi:10.1091/mbc.e05-04-0281. 16000372 PMC1196342

[15] Tonkin ML, Roques M, Lamarque MH, Pugniere M, Douguet D, Crawford J, Lebrun M, Boulanger MJ. 2011. Host cell invasion by apicomplexan parasites: insights from the co-structure of AMA1 with a RON2 peptide. Science 333:463–467. doi:10.1126/science.1204988. 21778402

[16] Lamarque M, Besteiro S, Papoin J, Roques M, Vulliez-Le Normand B, Morlon-Guyot J, Dubremetz JF, Fauquenoy S, Tomavo S, Faber BW, Kocken CH, Thomas AW, Boulanger MJ, Bentley GA, Lebrun M. 2011. The RON2-AMA1 interaction is a critical step in moving junction-dependent invasion by apicomplexan parasites. PLoS Pathog 7:e1001276. doi:10.1371/journal.ppat.1001276. 21347343 PMC3037350

[17] Tyler JS, Boothroyd JC. 2011. The C-terminus of Toxoplasma RON2 provides the crucial link between AMA1 and the host-associated invasion complex. PLoS Pathog 7:e1001282. doi:10.1371/journal.ppat.1001282. 21347354 PMC3037364

[18] Fritz HM, Buchholz KR, Chen X, Durbin-Johnson B, Rocke DM, Conrad PA, Boothroyd JC. 2012. Transcriptomic analysis of toxoplasma development reveals many novel functions and structures specific to sporozoites and oocysts. PLoS One 7:e29998. doi:10.1371/journal.pone.0029998. 22347997 PMC3278417

[19] Kim K. 2018. The epigenome, cell cycle, and development in Toxoplasma. Annu Rev Microbiol 72:479–499. doi:10.1146/annurev-micro-090817-062741. 29932347

[20] Balaji S, Babu MM, Iyer LM, Aravind L. 2005. Discovery of the principal specific transcription factors of Apicomplexa and their implication for the evolution of the AP2-integrase DNA binding domains. Nucleic Acids Res 33:3994–4006. doi:10.1093/nar/gki709. 16040597 PMC1178005

[21] Licausi F, Ohme-Takagi M, Perata P. 2013. APETALA2/ethylene responsive factor (AP2/ERF) transcription factors: mediators of stress responses and developmental programs. New Phytol 199:639–649. doi:10.1111/nph.12291. 24010138

[22] Campbell TL, De Silva EK, Olszewski KL, Elemento O, Llinas M. 2010. Identification and genome-wide prediction of DNA binding specificities for the ApiAP2 family of regulators from the malaria parasite. PLoS Pathog 6:e1001165. doi:10.1371/journal.ppat.1001165. 21060817 PMC2965767

[23] De Silva EK, Gehrke AR, Olszewski K, Leon I, Chahal JS, Bulyk ML, Llinas M. 2008. Specific DNA-binding by apicomplexan AP2 transcription factors. Proc Natl Acad Sci USA 105:8393–8398. doi:10.1073/pnas.0801993105. 18541913 PMC2423414

[24] Jeninga MD, Quinn JE, Petter M. 2019. ApiAP2 transcription factors in Apicomplexan parasites. Pathogens 8:47. doi:10.3390/pathogens8020047. 30959972 PMC6631176

[25] Iwanaga S, Kaneko I, Kato T, Yuda M. 2012. Identification of an AP2-family protein that is critical for malaria liver stage development. PLoS One 7:e47557. doi:10.1371/journal.pone.0047557. 23144823 PMC3492389

[26] Kafsack BF, Rovira-Graells N, Clark TG, Bancells C, Crowley VM, Campino SG, Williams AE, Drought LG, Kwiatkowski DP, Baker DA, Cortes A, Llinas M. 2014. A transcriptional switch underlies commitment to sexual development in malaria parasites. Nature 507:248–252. doi:10.1038/nature12920. 24572369 PMC4040541

[27] Painter HJ, Campbell TL, Llinas M. 2011. The Apicomplexan AP2 family: integral factors regulating Plasmodium development. Mol Biochem Parasitol 176:1–7. doi:10.1016/j.molbiopara.2010.11.014. 21126543 PMC3026892

[28] Yuda M, Iwanaga S, Shigenobu S, Kato T, Kaneko I. 2010. Transcription factor AP2-Sp and its target genes in malarial sporozoites. Mol Microbiol 75:854–863. doi:10.1111/j.1365-2958.2009.07005.x. 20025671

[29] Zhang C, Li Z, Cui H, Jiang Y, Yang Z, Wang X, Gao H, Liu C, Zhang S, Su XZ, Yuan J. 2017. Systematic CRISPR-Cas9-mediated modifications of Plasmodium yoelii ApiAP2 genes reveal functional insights into parasite development. mBio 8. doi:10.1128/mBio.01986-17. PMC5727417 29233900

[30] Huang S, Holmes MJ, Radke JB, Hong DP, Liu TK, White MW, Sullivan WJ, Jr. 2017. Toxoplasma gondii AP2IX-4 regulates gene expression during bradyzoite development. mSphere 2 doi:10.1128/mSphere.00054-17. PMC5352832 28317026

[31] Hong DP, Radke JB, White MW. 2017. Opposing transcriptional mechanisms regulate Toxoplasma development. mSphere 2. doi:10.1128/mSphere.00347-16. PMC5322347 28251183

[32] Radke JB, Worth D, Hong D, Huang S, Sullivan WJ, Jr, Wilson EH, White MW. 2018. Transcriptional repression by ApiAP2 factors is central to chronic toxoplasmosis. PLoS Pathog 14:e1007035. doi:10.1371/journal.ppat.1007035. 29718996 PMC5951591

[33] Walker R, Gissot M, Croken MM, Huot L, Hot D, Kim K, Tomavo S. 2013. The Toxoplasma nuclear factor TgAP2XI-4 controls bradyzoite gene expression and cyst formation. Mol Microbiol 87:641–655. doi:10.1111/mmi.12121. 23240624 PMC3556193

[34] Radke JB, Lucas O, De Silva EK, Ma Y, Sullivan WJ, Jr, Weiss LM, Llinas M, White MW. 2013. ApiAP2 transcription factor restricts development of the Toxoplasma tissue cyst. Proc Natl Acad Sci USA 110:6871–6876. doi:10.1073/pnas.1300059110. 23572590 PMC3637731

[35] Wang J, Dixon SE, Ting LM, Liu TK, Jeffers V, Croken MM, Calloway M, Cannella D, Hakimi MA, Kim K, Sullivan WJ, Jr. 2014. Lysine acetyltransferase GCN5b interacts with AP2 factors and is required for Toxoplasma gondii proliferation. PLoS Pathog 10:e1003830. doi:10.1371/journal.ppat.1003830. 24391497 PMC3879359

[36] Farhat DC, Swale C, Dard C, Cannella D, Ortet P, Barakat M, Sindikubwabo F, Belmudes L, De Bock PJ, Coute Y, Bougdour A, Hakimi MA. 2020. A MORC-driven transcriptional switch controls Toxoplasma developmental trajectories and sexual commitment. Nat Microbiol 5:570–583. doi:10.1038/s41564-020-0674-4. 32094587 PMC7104380

[37] Walker R, Gissot M, Huot L, Alayi TD, Hot D, Marot G, Schaeffer-Reiss C, Van Dorsselaer A, Kim K, Tomavo S. 2013. Toxoplasma transcription factor TgAP2XI-5 regulates the expression of genes involved in parasite virulence and host invasion. J Biol Chem 288:31127–31138. doi:10.1074/jbc.M113.486589. 24025328 PMC3829425

[38] Lesage KM, Huot L, Mouveaux T, Courjol F, Saliou JM, Gissot M. 2018. Cooperative binding of ApiAP2 transcription factors is crucial for the expression of virulence genes in Toxoplasma gondii. Nucleic Acids Res 46:6057–6068. doi:10.1093/nar/gky373. 29788176 PMC6159514

[39] Waldman BS, Schwarz D, Wadsworth MH, 2nd, Saeij JP, Shalek AK, Lourido S. 2020. Identification of a master regulator of differentiation in Toxoplasma. Cell 180:359–372.e16. doi:10.1016/j.cell.2019.12.013. 31955846 PMC6978799

[40] Cooper DG, Fassler JS. 2019. Med15: glutamine-rich mediator subunit with potential for plasticity. Trends Biochem Sci 44:737–751. doi:10.1016/j.tibs.2019.03.008. 31036407

[41] Oehring SC, Woodcroft BJ, Moes S, Wetzel J, Dietz O, Pulfer A, Dekiwadia C, Maeser P, Flueck C, Witmer K, Brancucci NM, Niederwieser I, Jenoe P, Ralph SA, Voss TS. 2012. Organellar proteomics reveals hundreds of novel nuclear proteins in the malaria parasite Plasmodium falciparum. Genome Biol 13:R108. doi:10.1186/gb-2012-13-11-r108. 23181666 PMC4053738

[42] Sidik SM, Huet D, Ganesan SM, Huynh MH, Wang T, Nasamu AS, Thiru P, Saeij JPJ, Carruthers VB, Niles JC, Lourido S. 2016. A genome-wide CRISPR screen in Toxoplasma identifies essential apicomplexan genes. Cell 166:1423–1435.e12. doi:10.1016/j.cell.2016.08.019. 27594426 PMC5017925

[43] Andenmatten N, Egarter S, Jackson AJ, Jullien N, Herman JP, Meissner M. 2013. Conditional genome engineering in Toxoplasma gondii uncovers alternative invasion mechanisms. Nat Methods 10:125–127. doi:10.1038/nmeth.2301. 23263690 PMC3605914

[44] Behnke MS, Radke JB, Smith AT, Sullivan WJ, Jr, White MW. 2008. The transcription of bradyzoite genes in Toxoplasma gondii is controlled by autonomous promoter elements. Mol Microbiol 68:1502–1518. doi:10.1111/j.1365-2958.2008.06249.x. 18433450 PMC2440561

[45] Fox BA, Rommereim LM, Guevara RB, Falla A, Hortua Triana MA, Sun Y, Bzik DJ. 2016. The Toxoplasma gondii rhoptry kinome is essential for chronic infection. mBio 7. doi:10.1128/mBio.00193-16. PMC4959664 27165797

[46] Hunt A, Russell MRG, Wagener J, Kent R, Carmeille R, Peddie CJ, Collinson L, Heaslip A, Ward GE, Treeck M. 2019. Differential requirements for cyclase-associated protein (CAP) in actin-dependent processes of Toxoplasma gondii. Elife 8. doi:10.7554/eLife.50598. PMC6785269 31577230

[47] Brown KM, Long S, Sibley LD. 2017. Plasma membrane association by N-acylation governs PKG function in Toxoplasma gondii. mBio 8. doi:10.1128/mBio.00375-17. PMC5414004 28465425

[48] Shen B, Brown K, Long S, Sibley LD. 2017. Development of CRISPR/Cas9 for efficient genome editing in Toxoplasma gondii. Methods Mol Biol 1498:79–103. doi:10.1007/978-1-4939-6472-7_6. 27709570

[49] Heaslip AT, Nishi M, Stein B, Hu K. 2011. The motility of a human parasite, Toxoplasma gondii, is regulated by a novel lysine methyltransferase. PLoS Pathog 7:e1002201. doi:10.1371/journal.ppat.1002201. 21909263 PMC3164638

[50] Xia N, Ye S, Liang X, Chen P, Zhou Y, Fang R, Zhao J, Gupta N, Yang S, Yuan J, Shen B. 2019. Pyruvate homeostasis as a determinant of parasite growth and metabolic plasticity in Toxoplasma gondii. mBio 10. doi:10.1128/mBio.00898-19. PMC6561023 31186321

[51] Shen B, Sibley LD. 2014. Toxoplasma aldolase is required for metabolism but dispensable for host-cell invasion. Proc Natl Acad Sci USA 111:3567–3572. doi:10.1073/pnas.1315156111. 24550496 PMC3948255

