# Supplementary material for: Nuclear Factor AP2X-4 Governs the Expression of Cell Cycle- and Life Stage-Regulated Genes and is Critical for Toxoplasma Growth
Source: Microbiol Spectr. 2022 Jun 23;10(4):e00120-22. doi: 10.1128/spectrum.00120-22 (PMC9430314; doi:10.1128/spectrum.00120-22)
Supplement: Supplemental file 1 — Fig. S1 and S2 and Table S1. Download spectrum.00120-22-s0001.pdf, PDF file, 4.6 MB [file spectrum.00120-22-s0001.pdf]

Supplemental Material for

Nuclear factor AP2X-4 governs the expression of cell cycle and life stage regulated genes and is critical for *Toxoplasma* growth

Jingwen Zhang [1, #], Fuqiang Fan [1, #], Lihong Zhang [1], Bang Shen [1, 2, 3 *]

[1] State Key Laboratory of Agricultural Microbiology, College of Veterinary Medicine, Huazhong Agricultural University, Wuhan, Hubei Province, 430070, People's Republic of China.

[2] Key Laboratory of Preventive Medicine in Hubei Province, Wuhan, Hubei Province, 430070, People's Republic of China.

[3] Hubei Hongshan Laboratory, Wuhan, Hubei Province, 430070, People's Republic of China.

[#] Equal contributions

* Corresponding author: Bang Shen

Phone number: +86-27-87281810; Fax number: +86-27-87280408

Email: shenbang@mail.hzau.edu.cn

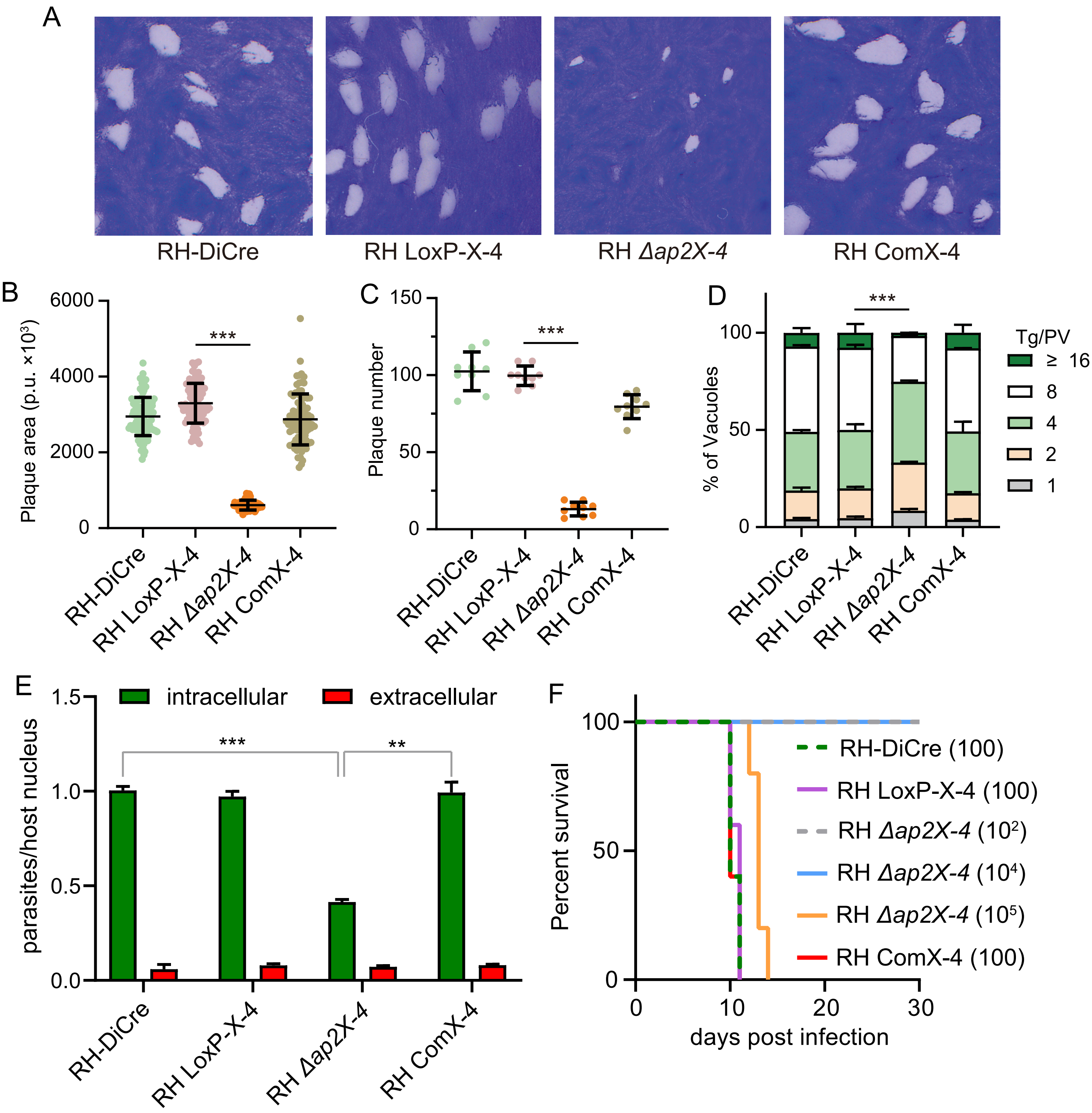

Figure S1. Assessment of the role of AP2X-4 in the type 1 strain RH-DiCre. A, Plaque assay comparing the overall growth of indicated strains. B-C, the sizes and numbers of plaques from A. Means ± SD, ****P* < 0.001, Student's t-test. D, Intracellular replication efficiency of indicated strains, as determined in Fig 3D after 24 hours of growth. Means ± SEM of three independent experiment. ****P* < 0.001, two-way ANOVA. E, Invasion efficiency of indicated strains into host cells, as done in Fig 5E. Means ± SD of three independent experiments, ****P* < 0.001, ***P* < 0.01,Student's t-test. F, survival curves of mice infected with indicated strains at different doses (100 - 10^5), as done in Fig 7A.

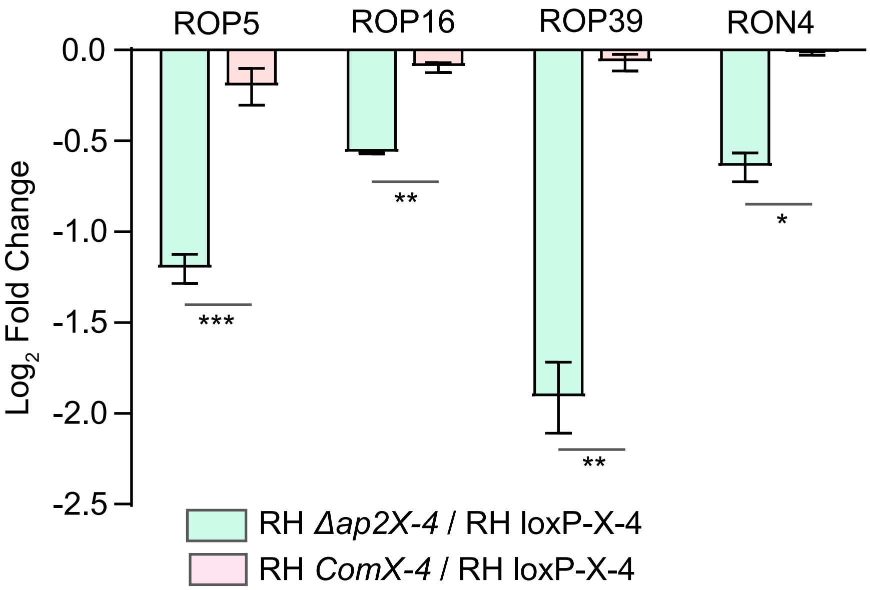

Figure S2. Comparison of transcript levels of selected genes in AP2X-4 modified strains, as determined by RT-PCR. Mean ± SEM of three independent experiments, $^{***}P < 0.001$, $^{**}P < 0.01$, $^{*}P < 0.05$, Student's t-test.

Table S1. Primers used in this study

Name	Sequence	Use
gRNA-AP2X4-Tag-Fw	GCAGCATCTAGAGTGTTTCG	To construct the CRISPR plasmid for tagging AP2X-4 with Ty or mAID
gRNA- AP2X4-Flox-Fw	GAGGAAACGACTAAGAGCAG	To construct the CRISPR plasmid to flox AP2X-4
gRNA−HXGPRT−Fw	TCAATGTAGGGCTTGCAGTG	To construct the CRISPR plasmid targeting *HXGPRT*
gRNA-CRISPR-Rv	CGACGGCCAGTGAATTCGAGGGGTCGAGCTACTATGGGAG	To construct locus specific CRISPR plasmids
5H-AP2X4-Ty-Fw	CGACGGCCAGTGAATTCGAGGAGAAGAACATTCGCAGCGG	Amplify 5'H for the Ty tagging construct
5H- AP2X4-Ty-Rv	CTGGTTCGTGTGGACCTCAAGTGCCGACTCCTGGCC	Amplify 5'H for the Ty tagging construct
3H- AP2X4-Ty-Fw	CACTTGCAGGATGAATTCCGCGTAAAGTATAGCAGGCAC	Amplify 3'H for the Ty tagging construct
3H- AP2X4-Ty-Rv	GCTATGACCATGATTACGCCCATAGCAGACATACGCGTG	Amplify 3'H for the Ty tagging construct
Ty-DHFR-Fw	GAGGTCCACACGAACCAG	Amplify Ty-DHFR for the Ty tagging construct
Ty-DHFR-Rv	GGAATTCATCCTGCAAGTG	Amplify Ty-DHFR for the Ty tagging construct
5'UTR-AP2X4-Fw	CGACGGCCAGTGAATTCGAGCCTTGTCTGCATCTGTTACCACG	Amplify 5'UTR of AP2X4 to construct the pAP2X4::DHFR plasmid
5'UTR-AP2X4-Rv	GATGTCTTCTGCGCGGGTTGCCTGCCGAGCCTCAGAGATAATC	Amplify 5'UTR of AP2X4 to construct the pAP2X4::DHFR plasmid
3'UTR-AP2X4-Fw	GCCACAAGTTCAGCGTGTCCGTGGAAAGAAAAGAGTGGAG	Amplify 3'UTR of AP2X4 to construct the pAP2X4::DHFR plasmid
3'UTR-AP2X4-Rv	GCTATGACCATGATTACGCCCATAGCAGACATACGCGTG	Amplify 3'UTR of AP2X4 to construct the pAP2X4::DHFR plasmid
Loxp-DHFR-loxp-Fw	CAACCCGCGCAGAAGACATC	Amplify DHFR to construct the pAP2X4::DHFR plasmid
Loxp-DHFR-loxp-Rv	GGACACGCTGAACTTGTGGC	Amplify DHFR to construct the pAP2X4::DHFR plasmid
pUC19-Fw	GGCGTAATCATGGTCATAGC	Amplify pUC19 vector to construct the AP2X4::DHFR plasmid, as well as the Ty/mAID tagging and floxing plasmids
pUC19-Rv	CTCGAATTCACTGGCCGTCG	Amplify pUC19 vector to construct the AP2X4::DHFR plasmid, as well as the Ty/mAID tagging and floxing plasmids
Tub8-Fw	TATCTCTGAGGCTCGGCAGGCGACGGCCAGTCTTAAGCTC	Amplify pTub8 for pLoxP-AP2X-4 construction

Tub8-Rv	GACATTTCAGCCATTTTGTCGGAATTCTATAACTTCGTATAA	Amplify pTub8 for pLoxP-AP2X-4 construction
AP2X4-Fw	AATGGCTGAAATGTCCTCG	Amplify AP2X-4 CDS for pLoxP-AP2X-4 construction
AP2X4-Rv	TTCGTGTGGACCTCAAGTGCCGACTCCTGG	Amplify AP2X-4 CDS for pLoxP-AP2X-4 construction
TY-YFP-DHFR-Fw	GAGGTCCACACGAACCAGG	Amplify TY-YFP-DHFR for pLoxP-AP2X-4 construction
TY-YFP-DHFR-Rv	CTCCACTCTTTTCTTTCCACGATTCCGTCAGCGGTCTGTC	Amplify TY-YFP-DHFR for pLoxP-AP2X-4 construction
pUC19/5H3H-Fw	GTGGAAAGAAAAGAGTGGAGAGAGA	Amplify backbone from pAP2X4::DHFR for pLoxP-AP2X-4 construction
pUC19/5H3H-Rv	CCTGCCGAGCCTCAGAGATA	Amplify backbone from pAP2X4::DHFR for pLoxP-AP2X-4 construction
UpAP2X4-Fw	TAGCGAGGGAGAGGCAATG	PCR1 to screen loxP-AP2X-4 clones
In-Tub-Rv	TTTGTCGGAATTCTATAACTTCGTATAA	PCR1 to screen loxP-AP2X-4 clones
InYFP-DHFR-Fw	GAGGTCGTGGGCTACGTCCC	PCR2 to screen loxP-AP2X-4 clones
DnAP2X4-Rv	CTCTGCTGCTCCGAGATAC	PCR2 to screen loxP-AP2X-4 clones
InAP2X4-Fw	CGATCCACATCAGTACCAG	PCR3 to screen loxP-AP2X-4 clones
InAP2X4-Rv	CTCCAAGTTGTGGAGAAGAC	PCR3 to screen loxP-AP2X-4 clones
ComX-4-CDS-Fw	GATCTAAAATGGTGAGCAAGATGGCTGAAATGTCCTCGG	To construct the *ComΔX-4* plasmid
ComX-4-CDS-Rv	GGCATAATCTGGAACATCGTAAGGATAAAGTGCCGACTCCTGGC	To construct the *ComΔX-4* plasmid
5"- In224050-Fw	CGATCCACATCAGTACCAG	PCR4 to screen *ComΔX-4* clones
3"-In224050-Rv	CTCCAAGTTGTGGAGAAGAC	PCR4 to screen *ComΔX-4* clones
In-HXGPRT-Fw	CCCATGTATATCCCCGACAAC	PCR5 to screen *ComΔX-4* clones
In-HXGPRT-Rv	CCGTTCAAGTGCACTTACC	PCR5 to screen *ComΔX-4* clones
5H-mAID-AP2X4-Fw	CGACGGCCAGTGAATTCGAGGAGAAGAACATTCGCAGCGG	Amplify 5’H to construct the mAID tagging plasmid
5H-mAID-AP2X4-Rv	TGCTAGCGCTCACCATCCTAGGAAGTGCCGACTCCTGGCC	Amplify 5’H to construct the mAID tagging plasmid
3H-mAID-AP2X4-Fw	AGCCTGAATGGCGAATGGGGCGTAAAGTATAGCAGGCAC	Amplify 3’H to construct the mAID tagging plasmid
3H-mAID-AP2X4-Rv	GCTATGACCATGATTACGCCCATAGCAGACATACGCGTG	Amplify 3’H to construct the mAID tagging plasmid
mAID-Fw	CCTAGGATGGTGAGCGCTAGCA	Amplify the mAID cassette to construct the mAID tagging plasmid
mAID-Rv	CCCATTCGCCATTCAGGCTG	Amplify the mAID cassette to construct the mAID tagging plasmid
RON2-RT-Fw	TGTCCGCAAGTTTGTGACCT	qRT-PCR of RON2

RON2-RT-Rv	TGAACAAGGCAAACGAACCG	qRT-PCR of RON2
RON4-RT-Fw	GCTCAGAGGTCGCGAAAAGA	qRT-PCR of RON4
RON4-RT-Rv	CTCGGCAGAGACTGTTCGAT	qRT-PCR of RON4
RON5-RT-Fw	AGTGCTTGCATGGATCACCA	qRT-PCR of RON5
RON5-RT-Rv	AGCGCGAGGTAAGTAAGAGC	qRT-PCR of RON5
Tubulin-RT-Fw	CACTGGTACACGGGTGAAGGT	qRT-PCR of β−tubulin
Tubulin-RT-Rv	ATTCTCCCTCTTCCTCTGCG	qRT-PCR of β−tubulin
ROP2A-RT-Fw	TGTGGTAGTTGGCAACCGTC	qRT-PCR of ROP2A
ROP2A-RT-Rv	CCGAATACTCGTGGAGTTGCT	qRT-PCR of ROP2A
ROP5-RT-Fw	TTGCTCAGCACTTCCGTAGG	qRT-PCR of ROP5
ROP5-RT-Rv	CCCATGAAACGAAGGGTCCA	qRT-PCR of ROP5
ROP16-RT-Fw	GATGATCGATGTCCACGGCT	qRT-PCR of ROP16
ROP16-RT-Rv	CAACCCTAAGGCCCACACAT	qRT-PCR of ROP16
ROP39-RT-Fw	GCCTCCTGTTACTGGGAACC	qRT-PCR of ROP39
ROP39-RT-Rv	CCACAGACGCCTCACGTATT	qRT-PCR of ROP39
